# National Black HIV/AIDS Awareness Day — February 7, 2017

**DOI:** 10.15585/mmwr.mm6604a1

**Published:** 2017-02-03

February 7 is National Black HIV/AIDS Awareness Day, an observance intended to raise awareness of human immunodeficiency virus (HIV) infection and acquired immunodeficiency syndrome (AIDS), and encourage action to reduce the disproportionate impact of HIV on blacks/African Americans (blacks) in the United States. From 2010 to 2014, the annual HIV diagnosis rate decreased for blacks by 16.2% (*1*); however, in 2015, blacks accounted for approximately half (45%) of all new HIV diagnoses (17,670), 74% of which were in men (*1*). The majority of these diagnoses were among gay and bisexual men.

The annual rate of HIV diagnosis among black women (26.2 per 100,000) was approximately 16 times the rate among white women (1.6) and approximately five times the rate among Hispanic women (5.3). Among blacks living with diagnosed HIV infection in 2013, 54% were receiving continuous HIV medical care (two or more CD4 or viral load tests ≥3 months apart) and 49% had a suppressed viral load (<200 copies/mL at most recent test) (*2*).

Additional information regarding National Black HIV/AIDS Awareness Day is available at https://www.cdc.gov/features/blackhivaidsawareness. Additional information about blacks and HIV is available at https://www.cdc.gov/hiv/group/racialethnic/africanamericans.

## References

[1] CDC. HIV surveillance report, 2015; vol. 27. Atlanta, GA: US Department of Health and Human Services, CDC; 2016. https://www.cdc.gov/hiv/library/reports/surveillance/

[2] CDC. Monitoring selected national HIV prevention and care objectives by using HIV surveillance data—United States and 6 dependent areas, 2014. HIV surveillance supplemental report 2016; vol. 21(no. 4). Atlanta, GA: US Department of Health and Human Services, CDC; 2016. https://www.cdc.gov/hiv/pdf/library/reports/surveillance/cdc-hiv-surveillance-supplemental-report-vol-21-4.pdf

